# “Feeling Like You Matter:” LGBTQ + Young Adult Perspectives on Affirmative Mental Healthcare

**DOI:** 10.1007/s11414-024-09919-x

**Published:** 2024-12-10

**Authors:** Marisa Mondave, Jessica Saleska, Jing Jing Wang, Elliot Bluma, Daynon Jackson, Yara Tapia, Leah Yashar, Bonnie T. Zima, Kristen R. Choi

**Affiliations:** 1 Department of Community Health Sciences, Fielding School of Public Health, UCLA, 650 Charles E Young Dr S, Los Angeles, CA 90095 USA; 2 Washington University at Saint Louis, 1 Brookings Dr, Saint Louis, MO 63130 USA; 3 Seattle’s LGBTQ+ Center, 400 E Pine St #100, Seattle, WA 98122 USA; 4 Seattle, WA USA; 5 Los Angeles, CA USA; 6 Los Angeles LGBT Center, 3055 Wilshire Blvd., Suite 360, Los Angeles, CA 90010 USA; 7 Jane and Terry Semel Institute for Neuroscience and Human Behavior, David Geffen School of Medicine, University of California Los Angeles (UCLA), 760 Westwood Plaza, Los Angeles, CA 90024 USA; 8 School of Nursing, University of California Los Angeles (UCLA), 700 Tiverton Dr, Los Angeles, CA 90095 USA; 9 Department of Health Policy and Management, Fielding School of Public Health, University of California Los Angeles (UCLA), 650 Charles E Young Dr S, Los Angeles, CA 90095 USA

## Abstract

The purpose of this study was to explore the experiences of LGBTQ + young people in mental healthcare and to understand their perspectives on what affirmative mental healthcare practice should look like. Between June and September of 2023, interviews were conducted with LGBTQ + young people ages 18–26 years in Los Angeles and Seattle. Interviews lasted 30 to 60 min and were transcribed verbatim for thematic analysis (*N* = 28). Four themes were developed from analysis of the interviews: (1) disconnection from community and self as an impetus for seeking formal mental healthcare; (2) marginalization during mental health service encounters; (3) the therapeutic power of belonging and mattering in the mental healthcare system; and (4) mutual human connection as the foundation for affirming mental healthcare experiences. Affirmative mental healthcare was defined by LGBTQ + young adults as free of judgment with priority placed on the therapeutic power of genuine human connection. The need for trans-specific mental healthcare was also highlighted. Participants also expressed a preference for providers with shared identities and experiences to alleviate feelings of isolation and increase feelings of connectivity. Mental health providers should emphasize connectivity and empathy to offer affirming care with LGBTQ + clients, such asking about client preferences for care, developing knowledge about sexuality and gender identity, and acknowledging provider limitations or knowledge gaps. Improving access to affirmative mental healthcare for LGBTQ + young adults (e.g., workforce development, training requirements) is needed to address the disparities in mental healthcare.

**Supplementary Information:**

The online version contains supplementary material available at 10.1007/s11414-024-09919-x.

## Introduction

An estimated 10% of adolescents (13 to 17 years) and transition age youth (18 to 26 years) in the United States (US) identify with a minority sexuality or gender category (lesbian/gay/bisexual/transgender/queer, LGBTQ +).^1^ This population is at significant risk for disparities in mental health outcomes compared to their cisgender and/or heterosexual counterparts, while also having poor access to mental health services.^2^ Lesbian, gay, and bisexual young people are two to four times as likely to develop a mental health disorder (e.g., depression, anxiety, posttraumatic stress) compared with heterosexual counterparts, and this risk is even higher for transgender young people.^3,4^ The risk gradient for suicide is similar, such that LGBTQ + individuals have two to four times higher risk for suicidality compared with their cisgender and/or heterosexual counterparts.^5^ Nearly half (42%) of LGBTQ + young people report considering suicide.^6,7^

Disparities in mental health and suicide among LGBTQ + young people are driven in part by inequitable access to mental health services. In a 2020 national survey of over 40,000 LGBTQ + individuals ages 13 to 24 years in the US, more than half (54%) reported wanting mental health services but not receiving any.^8^ A recent study reported that in 2016, only 13% of mental health facilities in the US offered LGBTQ + -specific treatment programs, suggesting significant gaps in available services.^9^ Specific barriers to mental health service access for this population include prior negative experiences with healthcare providers, and a perception that providers will not understand mental health needs related to sexual and gender identity.^8^ In addition to these interpersonal factors, there are also structural factors that contribute to disparities in mental healthcare access and outcomes for LGBTQ + young people, including societal stigma or exclusion and policies restricting access to care.^10–12^

In light of these pervasive mental health disparities and barriers to care, leading professional health organizations (e.g., American Academy of Pediatrics, American Academy of Nursing, American Academy of Child and Adolescent Psychiatry, Substance Abuse and Mental Health Services Administration [SAMHSA]) have strongly recommended LGBTQ-affirmative approaches to care delivery and strategies to increase access to mental healthcare for this population.^13–16^ Affirmative care is “an approach to health care delivery in which organizations, programs, and providers recognize, validate, and support the identity stated or expressed by the individuals served.”^17,18^ In mental health settings, offering affirmative care is especially important for LGBTQ + young people because providers are well-positioned to provide emotional support and validate feelings underpinning psychologic distress which may include affirming their identities.

Specific strategies for how providers can provide such affirmation in mental healthcare are not well understood, as there is limited empirical research on affirmative care in the mental health sector. A recent review of affirmative care interventions for transgender and gender expansive youth experiencing gender dysphoria identified four established affirmative care interventions (social transition, puberty blockers, hormone therapy, gender-affirmation surgery), but none of these explicitly addressed mental healthcare.^19^ Prior studies of affirmative care in mental health have focused on documenting barriers to affirming care and drivers of mental health and healthcare disparities for LGBTQ + individuals, such as the lack of provider knowledge, over-reliance on a medical model of mental health, provider bias and stigma, and insufficient access to services.^18,20–23^ While understanding barriers is important, there has been less emphasis on understanding facilitators of affirmative mental healthcare or operationalizing affirmative care in mental health practice.^24^ Understanding how affirmative care principles should be operationalized in mental healthcare delivery and the preferences of LGBTQ + individuals for affirming approaches to their mental health may contribute to reduced disparities in mental health outcomes and service access for this population.

The purpose of this qualitative research study was to explore lived experience of barriers to affirming mental healthcare and to understand the perspectives of LGBTQ + young people on what affirmative mental healthcare practice should look like. The study utilized Community-Partnered Participatory Research (CPPR) principles,^25^ partnering with community-based organizations in Los Angeles and Seattle throughout the study to center historically marginalized voices in the research process.

## Methods

### Design

This was a qualitative study conducted using principles of CPPR and thematic analysis to identify, establish, and maintain partnerships with LGBTQ + community-based organizations throughout the research process.^25,26^ CPPR is a recommended strategy for researchers to build trust with and empower the voices of historically marginalized communities by building upon existing community knowledge.^25,27^ The study applied the principles of power-sharing and two-way capacity development with community partners, two community-based LGBTQ + centers in Los Angeles and Seattle.^27^ Semi-structured interviews were conducted with LGBTQ + young adults in Los Angeles and Seattle who had received formal mental health services in the past from May to September 2023. Study procedures, including the informed consent process, were approved by the Institutional Review Board at UCLA.

### Sample

LGBTQ + young adults ages 18 to 26 years were the target population for this study. Participants were eligible for study inclusion if they had a history of receiving at least one type of formal mental healthcare (e.g., therapy, residential treatment, inpatient or partial hospitalization, psychiatric urgent care, other mental health visits), were not experiencing a mental health crisis at the time of the interview, and spoke English or Spanish. Participants were excluded if they had never received formal mental healthcare or did not identify with any LGBTQ + category. Forty-three individuals expressed interest in study participation and were screened for eligibility. Of these, 14 individuals were either ineligible to participate or unresponsive to follow-up efforts. Twenty-nine eligible individuals completed a study interview. All participants received a $75 gift card incentive for their time.

### Procedures

A semi-structured interview guide was developed to capture past experiences of mental healthcare and views on affirmative mental healthcare. Questions were co-developed by research team members and community partners; community partners reviewed the interview guide and provided feedback to best reflect community needs. LGBTQ + young adults were recruited through flyers, walk-in interview opportunities, and word of mouth at partner organizations. Interviews were conducted by the first author (MM) and lasted from 30 min to 1 h in person, by phone, and by the Internet depending on the preference of the participants. All interviews were audio recorded and transcribed using digital transcription software. Following interviews, the transcripts were checked against recordings and corrected to ensure accuracy prior to data analysis by the first author and a research assistant. Participant identifiers were removed throughout each transcript at this stage. Out of the 29 interviews conducted, 18 interviews were conducted with Los Angeles participants, and 11 interviews were with Seattle participants. One transcript was excluded from data analysis due to poor audio quality and inability to confirm transcription (final *N* = 28).

Researchers considered the ways in which interactions with participants might be influenced by the researchers’ personal and professional backgrounds throughout the research process. Reflexivity memos were recorded after each interview to note biases, assumptions, and reactions to each interview and to reduce unintended interviewer projection.^28^ The team members most closely involved in interviews (MM and KC) met weekly during data collection to reflect on interviews, discuss methodological adjustments, and consider biases or assumptions that might affect data collection.

### Data Analysis

A qualitative thematic analysis was conducted based on a six-phase approach, applying CPPR methods and conducting analysis through a series of collaborative researcher/community data analysis meetings.^26,31,32^ First, transcripts were read for data familiarization by two members of the research team (MM and EC). Second, interview text was systematically coded using process coding with gerunds to focus on the action of the participant, with independent coding of each interview by two analysts using the software, Atlas.ti, to minimize researcher bias (MM and KC).^32–34^ After initial coding, codes were reviewed and grouped into clusters, prioritizing codes that were salient to the research question, most frequently occurring, and most significant (all authors).^26^ To refine code clusters, codes and data were compared to identify cluster fit or the need for formation of a new cluster.^26^ Researchers and community partners worked collaboratively at this stage to combine small or similar clusters and divide large or complex clusters.^25^ Third, through a comparative analysis of codes in clusters, patterns of action or meaning in the data were identified to form candidate themes.^26^ Fourth, candidate themes were compared against one another to further combine or divide meaningful units of data; and then candidate themes were compared against codes and memos to finalize major attributes of each theme.^26^ Fifth, final themes were selected, refined, and named collaboratively by the research team and community partners to depict main themes.^26^ At this stage, researchers engaged an LGBTQ + youth artist from one study site to further interpret study findings by developing illustrations of themes. The use of visual data and arts in qualitative research (e.g., photography, collage) can be an effective strategy for youth and community engagement in research,^29,30^ and thus, the study incorporated a visual depiction of themes as a form of participatory data representation. Participant quotes are accompanied by a study label number in brackets.

## Results

A diverse range of identities were captured in this study as participants self-identified as holding multiple marginalized identities, shown in Table 1. From the 28 interviews included in analysis, four themes were developed: (1) the lack of LGBTQ + -affirming community environments and disconnection from self as a driver of formal mental healthcare help-seeking; (2) marginalization during mental health service encounters; (3) promoting belonging and mattering in the mental healthcare system; and (4) mutual human connection and compassion as the foundation for affirming mental healthcare experiences. Visual representations of themes are shown in Supplement 1.

**Table 1:** Participant demographics

	*N*	%
Sexual and gender identities		
Ace spectrum/asexual	4	14.3%
Bisexual	4	14.3%
Cisgender	2	7.1%
Gay	3	10.7%
Lesbian	2	7.1%
Non-binary	9	32.1%
Pansexual	5	17.9%
Queer	10	35.7%
Trans-masc	5	17.9%
Transgender	4	14.3%
Racial and ethnic identities		
Asian	5	17.9%
Black	11	39.3%
Hispanic/Latinx	5	17.9%
Multiracial	4	14.3%
Native American/Alaskan Native and other identities	3	10.7%
White	9	32.1%
Identified pronouns		
He/him	13	46.4%
She/her	9	32.1%
They/them	11	39.3%

Participants were asked to self-identify with the above demographic categories in an open-ended question. Many participants had more than one identity in each category; thus, frequencies may exceed the sample size of 28

### The Lack of LGBTQ + -Affirming Community Environments and Disconnection from Self as a Driver of Formal Mental Healthcare Help-Seeking

Participants described a lack of social support and othering within their social support systems as a catalyst for experiencing mental health issues and seeking mental healthcare. Descriptions of lack of social support and othering noted by participants included “feeling out of place with friends at school” [3], “put into silence” [7], “always had to hide it [sexuality]” [1], and “not fitting the mold” [7]. A participant reflected on the rural community they grew up in and stated, “There weren’t a lot of other people there where I lived, who I saw my identities reflected in…there definitely was not any queer or trans person that I knew was out” [24]. ‬‬One participant described their “sexualities being made fun of by my, siblings, by my, even my own mother” [7]‬, while another noted “Kids can be cruel… there were times where like, I was not treated very well” [28]. There were multiple participants who described serious stressors in their family and community environments, including homelessness, sexual assault, family mental illness, religious trauma, generational trauma, suicide attempts, immigration or acculturation stress, financial stress, and child abuse precipitating a need for mental healthcare. One participant noted their painful experience of family rejection: ‬‬‬‬‬‬‬‬‬‬‬‬‬‬‬‬‬‬‬‬‬‬‬‬‬‬‬‬‬‬‬‬‬‬‬‬‬‬‬‬‬‬‬‬‬‬‬‬‬‬‬‬‬‬‬‬‬‬‬‬‬‬‬‬‬‬‬‬‬‬‬‬‬‬‬‬‬‬‬‬‬‬‬‬‬‬‬‬‬‬‬‬‬‬‬‬‬‬‬‬‬‬‬‬‬‬‬‬‬‬‬‬‬‬‬‬‬‬‬‬‬‬

The guidance counselor told my mother and then I came home my mother was like really upset at me… she threw me to the back of the door and she was like, well, maybe you should kill yourself. You know, she—she called me a faggot that day. And so like after that, like I went into a deep, deep like silence like and after like five years I wasn’t crying…[7]

Several participants noted that exclusion, the lack of support, and marginalization could occur even within the queer community. This “gatekeeping” [21] was described by one participant as:

It’s so frequent that, like, the feeling of like loneliness, especially because of all the gatekeeping in the LGBT communities. You don’t ever really feel like… there’s one cohesive, coherent, like, community under one label [21].

Another participant observed, “Whatever they do at the LGBT center is like, for specific people, I guess. And it’s like, I’m not one of those people.”

As participants described feelings of disconnection from their social environment, participants discussed uncertainty about their identities or a disconnection from their sense of self resulting from these marginalizing, exclusionary, or stigmatizing social experiences. A participant described their internal state when they ultimately decided to seek formal mental healthcare as, “I was trying college and I had to drop out because I just couldn’t handle all the stuff that was going on in my head on my own‬” [19]. Another participant described exploring their sexuality as “confusing” [7] in a non-affirming environment while in college. In describing multiple depressive episodes leading up to formal help-seeking, a participant recounted “going through that cycle time and time again and feeling really unsupported and not knowing how to support or show up for myself‬” [22]. Many participants noted the role of intersecting identities compounding challenges seeking mental healthcare, such as one participant who said “I just couldn’t add to the pile of minoritized identities and stuff I was going through when I was younger” [19]. Another participants described internal barriers to seeking care resulting from stigmatizing experiences in their communities as, “self-procrastination, body dysmorphia, like all these, these little things that that just that hold me back from being able to do what I want to do” [2]. ‬‬‬‬‬‬‬‬‬‬‬‬‬‬‬‬‬‬‬‬‬‬‬‬‬‬‬‬‬‬‬‬‬‬‬‬‬‬‬‬‬‬‬‬‬‬‬‬‬‬‬‬‬‬‬‬‬‬‬‬‬‬‬‬‬‬‬‬‬‬‬‬‬‬‬‬‬‬‬‬‬‬‬‬‬‬‬‬‬‬‬‬‬

### Marginalization during Mental Health Service Encounters

#### Subtheme: Structural Competency Disparities Among Mental Healthcare Providers

In addition to experiencing marginalization and stigma in their families or communities, participants noted these same issues occurring during formal mental healthcare encounters when they eventually decided to seek care. Negative care experiences were sometimes interpersonal and sometimes structural in how participants interacted with providers and systems of care. Participants identified negative experiences with mental healthcare providers including minimizing, misinformation, judgment, disrespecting pronouns, reinforcing a gender binary, the lack of provider competency, the lack of provider knowledge, and provider identity misalignment. One participant shared their experience with receiving misinformation from a provider on medically transitioning after initially opening up to them about their trans identity:

And I was like, leading up to me, figuring out I thought that I was trans and like finding the language for it and finding online like virtual community faces for it… she was actually the first person that I came out to… She also was like, ‘Are you sure? Think about it, that’s irreversible,’ which is wrong, it’s actually reversible. The first person that I told was receiving it in a way that made me feel unworthy of care [24].‬‬‬‬‬‬‬‬‬‬‬‬‬‬‬‬‬‬‬‬‬‬‬‬‬‬‬‬‬‬‬‬‬‬‬‬

Multiple participants feared opening up to their provider about their identities and experiences. One participant expressed that “there was a time where I just straight up lied about what I was going through. And the reason for that is again, because I just felt like…I don’t know if I can trust you and it’s easier to talk about things that are made up to gauge reactions and safety” [21]. The participant later expressed “it felt like a thing where if I brought up being queer, it would just get minimized in some way” [21]. One participant even expressed their experience feeling a lack of safety in the client-therapist relationship, “the therapist would bring their own values into the conversation and that immediately made it an unsafe space to discuss [sexual orientation and gender identity]” [3]. Multiple participants noted a lack of competency in gender and sexuality among mental health providers and felt that they had no choice but to educate their providers on queer identity as it relates to mental health. A participant noted, “I was kind of put in a position where I felt like I had to educate my therapist about my own mental health” [27]. Likewise, in recounting a positive care experience with a queer mental health provider, a participant shared, “I don't have to like‬, stop to explain, where I’m coming from with a lot of queer and trans issues I’m facing, which I appreciate. I don’t like having to educate my providers… I really hate educating providers. Like I’m just giving you a free service at this point” [29].

### Subtheme: Structural Barriers to LGBTQ + Affirming Mental Healthcare

Participants identified several structural or systemic barriers that inhibited timely receipt of care and contributed to feelings of marginalization in the mental healthcare system. Structural barriers included delays in accessing care, long waitlists, involuntary care, insurance barriers, financial barriers, age restrictions, language barriers, medical mistrust, difficulties finding care in one’s geographic area, and proximity to care. Regarding affordability of care, one participant noted, “Unless you have like a really well-paying job with good health insurance, it’s very hard for therapy to be sustainable financially for most of the queer people I know” [22]. Some participants were unable to use their parent’s insurance for mental health services due to unsupportive relationships or beliefs related to mental health, such as one participant who said, “My parents don't believe in therapy” [24]. Another shared the belief of mental health as a taboo at home, stating “When it comes to mental health and things like that, it really wasn't a thing that was talked about a lot at home” [7]. Frustration with long wait times to receive care and marginalization in the mental health system, even within organizations that were affirming, were significant sources of difficulty One participant expressed, “Being on a waiting list is… just anxious or like tedious or something whenever you need like immediate help” [11], while another described how “you keep getting knocked back down the waiting lists” [2] as they had waited 6 months before an initial consultation. Other participants expressed a complete lack of any affirmative care available nearby. One participant expressed that they had to drive “four hours or five hours” [24] to another state to receive therapy related to their gender identity. Another stated, “There’s like no care in [town name redacted] that supports trans services” [13]

### Promoting Belonging and Mattering in the Mental Healthcare System 

Participants expressed a need for both belonging and mattering within the mental healthcare system, and how much more effective they experienced mental healthcare to be when these factors were present. In reflecting on a positive and affirming mental healthcare experience, a participant defined ideal mental healthcare as “being able to feel like you matter, and your thoughts and feelings matter. It’s not about you know, feeding the things that are bothering you” [18]. ‬‬When participants reflect on mental healthcare providers or encounters that were affirming and contributed to the feeling that they mattered and belong, participants described these interactions or providers as “understanding” [25], “good at listening” [18], “being heard” [24], “non judgmental curiosity” [22], “respectful” [6], “get[ting] treated with dignity” [18], being “trustworthy” [18], not “assuming ill intent” [18], and “treated like my problems are solvable” [18]. A participant shared of an affirming provider, “It makes all the difference, being able to…[have] someone try and take care of the problems that are actually bothering you rather than trying to fix things that aren't a problem” [18]. Another stated, “She was understanding, very understanding. She helped me understand a couple of things that I myself felt like I was lost on and it actually made me…feel okay‬” [5].‬‬‬‬‬‬‬‬‬‬‬‬‬‬‬‬‬‬‬‬‬‬‬‬‬‬‬‬‬‬‬‬‬‬‬‬‬‬‬‬‬‬‬‬‬‬‬‬‬‬‬‬‬‬‬‬‬‬‬‬‬‬‬‬‬‬‬‬‬‬‬‬‬‬‬‬‬‬‬

Participants emphasized normalizing their experiences and offering practical tools and supports led to care experiences where they felt that they mattered. For example, one participant described a mental health provider who “would oftentimes tell me stories from other clients with no, you know, identifying information of course, but from other clients that were similar or based in the same problems”‬ [19] as normalizing their experience. Another described “being on the same wavelength” [18] with a provider. Practical recommendations for affirming mental healthcare shared across participants are shown in Table 2. Participants emphasized the importance of mental health encounters characterized by empathetic listening skills free of judgment; the application of relevant knowledge about gender identity and sexual orientation to mental health issues; trauma-informed care; and attention to intersectionality (e.g., race/ethnicity, disability, neurodivergence).

**Table 2:** Affirmative mental healthcare recommendations for providers and organizations

Recommendations for providers • Introduce self and share identities • Ask for clients’ pronouns and preferred name • Use clients’ pronouns and preferred names in all interactions; if accidental misgendering occurs: apologize, affirm, and move on • Avoid centering all mental health conversations around gender or sexuality—allow the client bring it up if they believe it is relevant • Ensure knowledge and competency when conversing on key topics that might be salient to the mental well-being of LGBTQ + clients: queer gender and sexuality, trans health, medical transitions, trauma, LGBTQ + history with healthcare • Acknowledge any provider limitations or knowledge gaps directly with the client • Ask consent to talk about highly sensitive topics • Check-in with client about how they feel about the care they are receiving • Signal comfortability hearing about sensitive topics (e.g., queer relationships, experiences with homelessness) • Check-in with client regularly outside of therapy sessions • Provide tools and skills for client to navigate on their own after care ends • Provide resources for medically transitioning, including gender-affirming medical care support letters when appropriate
Recommendations for mental health organizations • Offer, require, or incentivize trainings for staff in key areas for developing knowledge and competency in key topics that are salient to the mental health of LGBTQ + clients: queer gender and sexuality, trans health, medical transitions, trauma, LGBTQ + history with healthcare • Prioritize a diversity of identities and experiences when hiring providers • Include visual symbols of being queer friendly in clinics and offices • Provide accessible information about care options and services, including directory of provider and service information (e.g., provider identity, specialties, ratings/reviews, costs) • Provide community spaces for affirmative care through support groups and education • Ensure that there are all-gender facilities available

### Mutual Human Connection and Compassion as the Foundation for Affirming Mental Healthcare Experiences

As participants reflect on what affirmative mental healthcare meant or would ideally look like, a common unifying theme among participant suggestions was the importance of authentic, equitable human connection and a sense of mutuality with providers. Compassionate relationships were often at the core of how participants defined the kind of affirming care they wished to receive during mental health encounters, as one participant noted when saying, “My definition of compassionate mental health care is when people treat you more like you’re equal, than you’re not”‬ [1]. One participant contrasted a “clinical” experience with a “relational” experience of mental healthcare, saying:

In that time, I started seeing another therapist. And this person, we also had shared identities. But their approach was like, fairly clinical… I bring that up to say that my long term therapist, I really liked her style of like, still unpacking the psychology of it, but it’s also like very relational. She’s very empathetic. She can hold complexity really well [22]‬.‬‬‬‬‬‬‬‬‬‬‬‬‬‬‬‬‬‬‬‬‬‬‬‬‬‬‬‬‬‬‬‬‬‬‬‬

Many participants called out the power dynamic that naturally occurs within mental healthcare, expressing a desire for this to change and for providers to create a shared sense of vulnerability. One participant spoke on this desire for a sense of shared vulnerability with their therapist:

I think what would be helpful is, kind of like… in any kind of relationship, just taking it slower in the sense of, I also want to learn more about my therapist and know the kinds of things that they have dealt with or that they have experience with [25].

There was a strong preference among participants for connectivity with providers via shared identities and experiences. Participants frequently expressed a preference to work with mental health providers who shared their queer identities, as well as those who had concordant racial/ethnic identities or spoke the same language. One participant explained this preference for shared identities with their provider (in this case, trans-masculine, or “trans-masc” for short) as a way to be understood and experience therapeutic connection: ‬‬‬‬‬‬‬‬‬‬‬‬‬‬‬‬‬‬‬‬‬‬‬‬‬‬‬‬‬‬‬‬‬‬‬‬‬

I feel like what would work best is someone who understands experiences similar to mine because they've like gone through them, like trans therapists. Specifically even like a trans-masc therapist, who understands what it’s like being trans masc versus like, a therapist, she just has an idea of what it’s like to be trans [3].

In discussing the importance of shared identity with mental health providers, trans-specific care was raised as a critical component of affirming mental healthcare. Participants described trans-specific care as care that respects gender identity and does not reinforce a gender binary (i.e., man/woman, male/female, masc/fem)with providers who are knowledgeable about information related to medical transitioning. Participants acknowledged that not all clinicians would necessarily hold a perfect set of shared identities and experiences with their LGBTQ + clients but emphasized the importance of flexibility and openness in these instances. One participant described having a provider with this quality of openness:

Somebody who is more open to the ways that gender can be expressed beyond…just the ways that you’re most familiar with, or even like beyond ways that are super comfortable [27].

Although affirming mental healthcare is experienced in part through interactions with providers, participants also noted a need for mental health system change in order for care to be received in an affirming context. These included addressing access barriers (e.g., cost, long waitlists, geographic location), more information about the availability of queer providers, and more LGBTQ+-specific care settings. Several participants expressed their comfortability and preference for seeking care via LGBTQ+ such as those who said, “I’m satisfied. I’m getting therapy from the LGBT center” [18], “It was super easy” [16], and “I’m seeing a therapist here at the LGBTQ center…so that’s been a lot better for me and a lot more affirming” [6]. Participants saw affirming provider actions and affirming mental health systems as interconnected, as one participant described the importance of education, activism, and advocacy by providers for system change:

I think people who are first of all educated on you know, ableism and racism and sexism and are activists, like, will advocate for their clientele. I think [this] is really important‬. I think you can be the kindest fucking therapist in the world, but if you’re not educated, like, on racism, it's like that tells me that you only care about specific clientele [29].‬‬‬‬‬‬‬‬‬‬‬‬‬‬‬‬‬‬‬‬‬‬‬‬‬‬‬‬‬‬‬‬‬‬‬‬

## Discussion

This qualitative study sheds light on the interplay between social, structural, and interpersonal factors influencing LGBTQ + young adults’ mental health experiences and preferences for affirmative care. The study found that LGBTQ + young adults commonly find themselves seeking mental healthcare because of non-affirming home or community environments, which may be inadvertently reinforced by marginalizing or disrespectful mental healthcare experiences. Affirmative mental healthcare experiences, on the other hand, were characterized by a sense of belonging and mattering in the mental healthcare system, experiencing mutual human connection with providers, and receiving compassionate care. These themes underscore the need for more accessible, affirmative, and culturally competent mental health services that address the unique challenges faced by LGBTQ + individuals, emphasizing the importance of genuine human connection and understanding in therapeutic relationships. Findings align with prior studies that highlight the disparity in access to mental healthcare among LGBTQ + young adults and barriers to seeking services including the lack of family support, financial barriers, past negative experiences with mental health clinicians, and perceived judgment surrounding queer identity from clinicians.^5^

The findings of this study further emphasize the feelings of loneliness, disconnection, and rejection LGBTQ + young adults’ experience which may be replicated by mental health providers. To address this gap in the quality of mental healthcare that LGBTQ + young adults receive, clinicians must reflect and address practices and interactions that allude judgment, minimization, and disrespect of queer identities. The study addresses the gap in defining affirmative mental healthcare that leading health organizations have called for.^13,15^ Participants defined affirmative mental healthcare as care that is free of judgment, emphasizes empathy and genuine human connection, alleviates feelings of isolation through shared vulnerability, and addresses the needs of transgender and gender expansive individuals. Affirmative mental healthcare for transgender and gender expansive individuals includes provider competency, respect towards pronouns, and comfort or willingness to explore gender outside of a gender binary. Clinicians who strive to provide affirmative mental healthcare should self-identify themselves as such to increase ease of accessibility and identification for potential LGBTQ + clients.

This study captured a diverse range of queer identities and intersecting racial/ethnic identities. Further studies should prioritize diversity in demographics as well as recruiting non- English speakers and LGBTQ + young adults living in varying regions across the US. Despite offering interviews in Spanish to enhance inclusivity, only English-speaking participants volunteered to participate in the study. Partnerships with community agencies in Los Angeles and Seattle allowed reach to participants residing in large metropolitan areas. This served to be both a strength and limitation in the study as a racially diverse participant pool was recruited, but there was under-representation of other groups such as LGBTQ + young adults living in rural areas. This research points to an opportunity to develop affirmative care interventions for mental health, as well as further investigation of the perspectives of LGBTQ + young adults on affirmative care with other intersectional characteristics and identities (e.g., disability, language, rurality).

## Implications for Behavioral Health

LGBTQ + young adults face complex challenges in accessing and receiving affirmative mental healthcare. While there is emerging evidence on affirmative care interventions, the mental health crisis in the US along with mental healthcare disparities for LGBTQ + young adults remain widely unaddressed. This study underscores the importance of genuine connection and understanding in therapeutic relationships. Mental healthcare that specifically targets the needs of transgender and gender expansive patients was also highlighted. Clinicians should consider the social, structural, and interpersonal contexts of their LGBTQ + clients in addition to personal history to best meet their needs. Strategies for affirmative care recommended by study participants can be implemented in behavioral health practice to achieve these goals, including asking about client preferences for care, developing knowledge about sexuality and gender identity, and acknowledging provider limitations or knowledge gaps openly. More broadly, it is important to prioritize diversifying the behavioral healthcare workforce in terms of sexuality and gender identity; to incentivize or require trainings for behavioral health clinicians on gender identity and sexuality; and to advocate for greater access to mental healthcare services for LGBTQ + young people.

## Supplementary Information

Below is the link to the electronic supplementary material.

Supplementary file1 (DOCX 2104 KB)

## Declarations

### Reporting Guidelines

COREQ
