# Supplementary material for: “Feeling Like You Matter:” LGBTQ + Young Adult Perspectives on Affirmative Mental Healthcare
Source: J Behav Health Serv Res. 2024 Dec 10;52(1):155–67. doi: 10.1007/s11414-024-09919-x (PMC11685268; doi:10.1007/s11414-024-09919-x)
Supplement: Supplementary file 1 — Supplementary file1 (DOCX 2104 KB) [file 11414_2024_9919_MOESM1_ESM.docx]

**Supplement 1: Visual Representation of Themes**

**Figure S1.** Illustration of “Disconnection from community and self as an impetus for seeking formal mental healthcare”

**
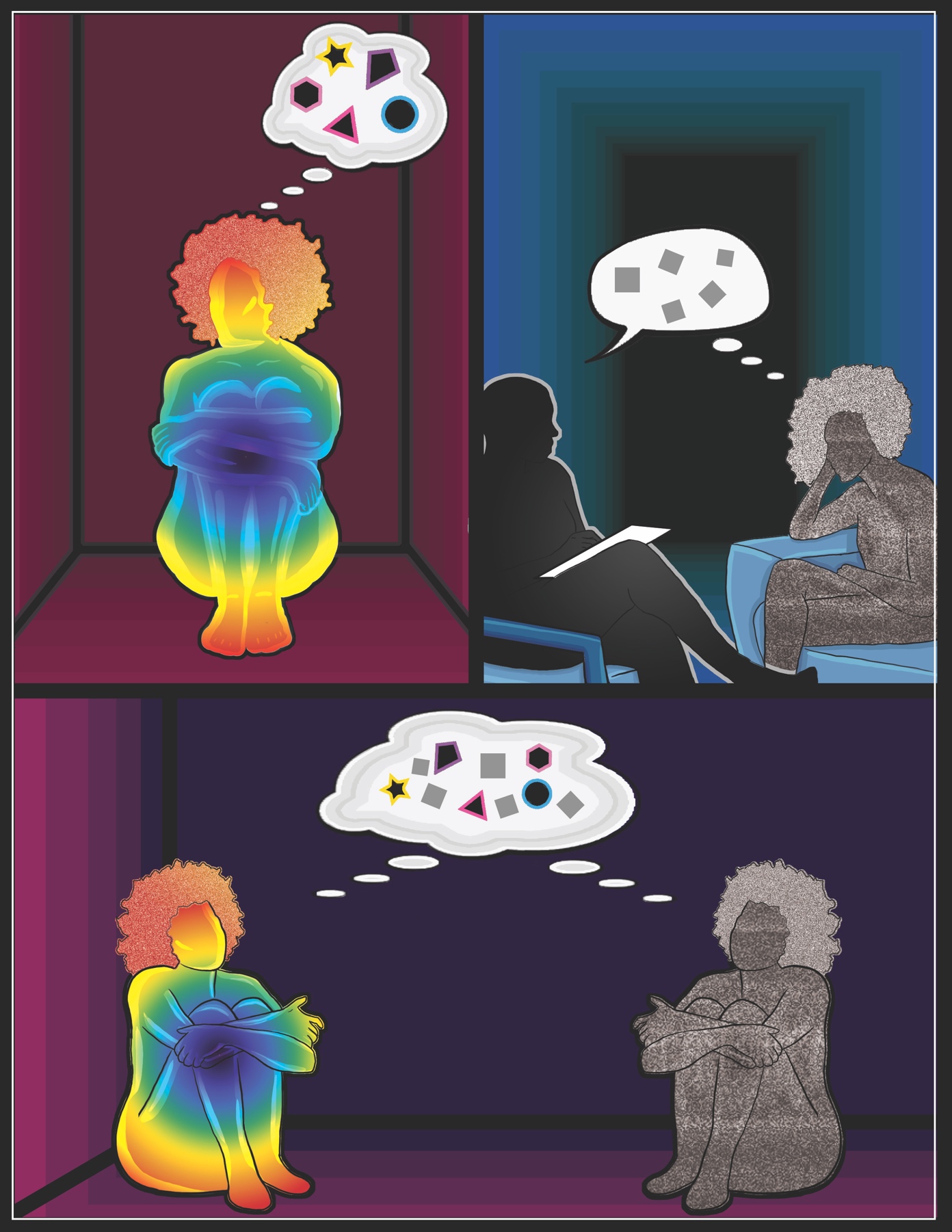
**

**Figure S2.** Illustration of “Marginalization during mental health service encounters”

**
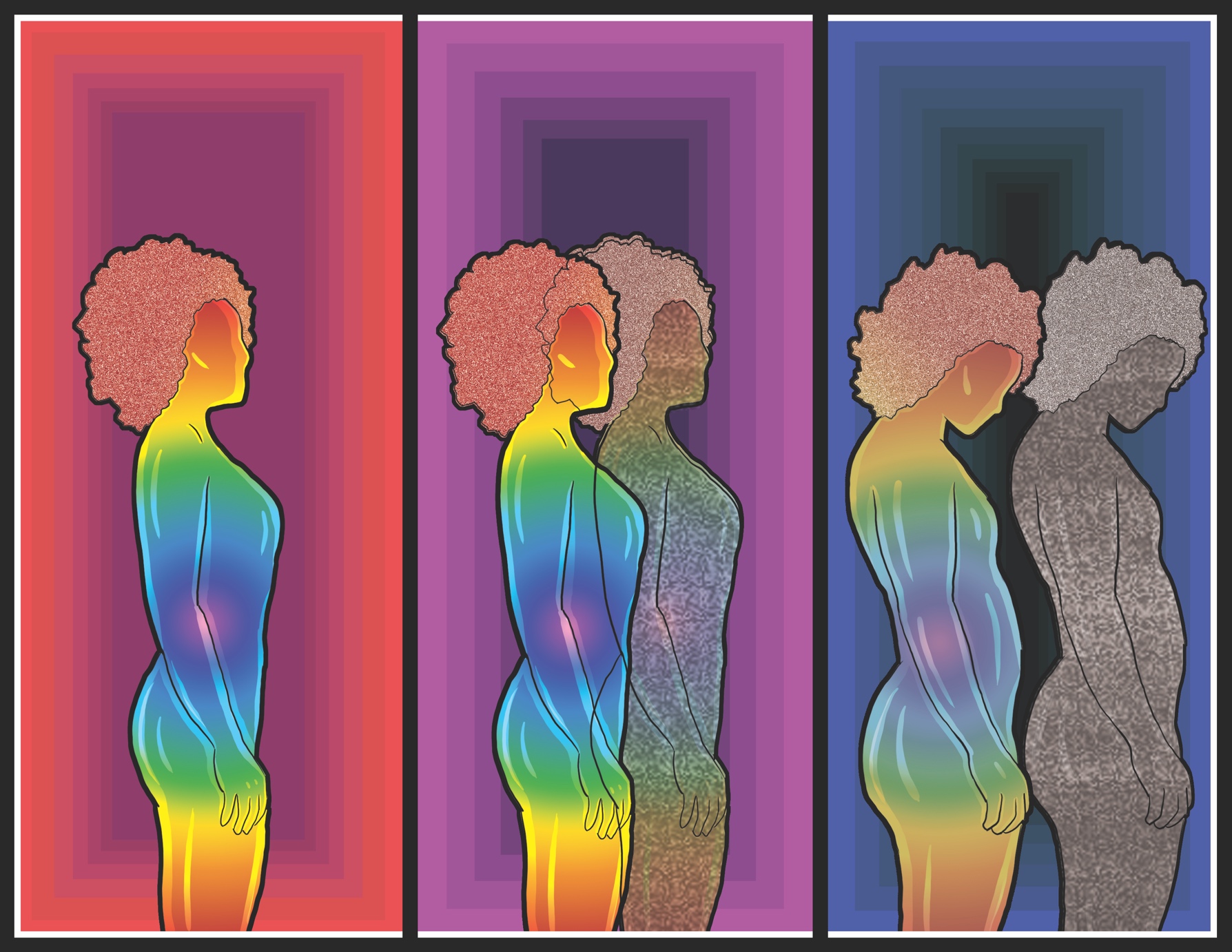
**

**Figure S3.** Illustration of “Therapeutic power of belonging and mattering in the mental healthcare system”

**
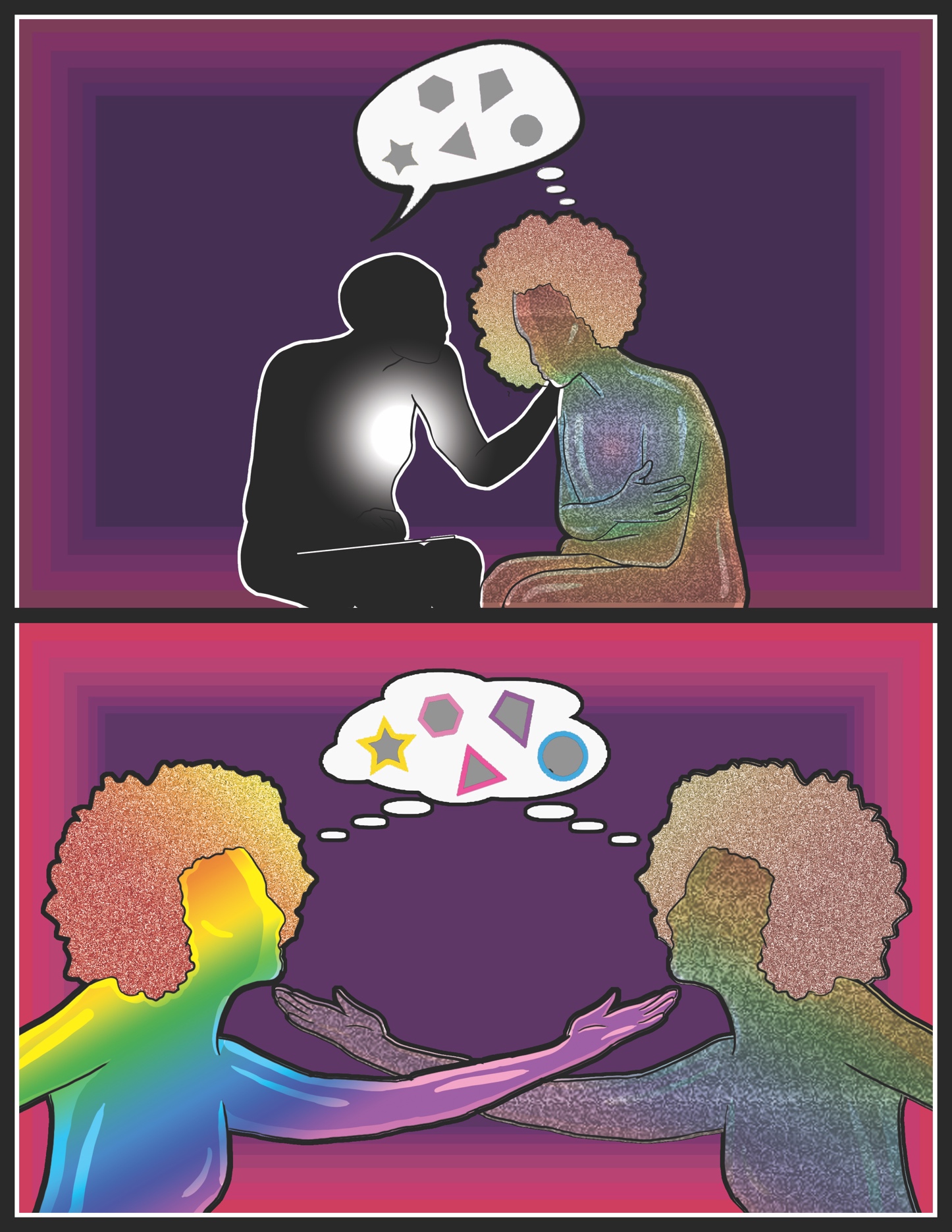
**

**Figure S4.** Illustration of “Mutual human connection as the foundation for affirming mental healthcare experiences” **
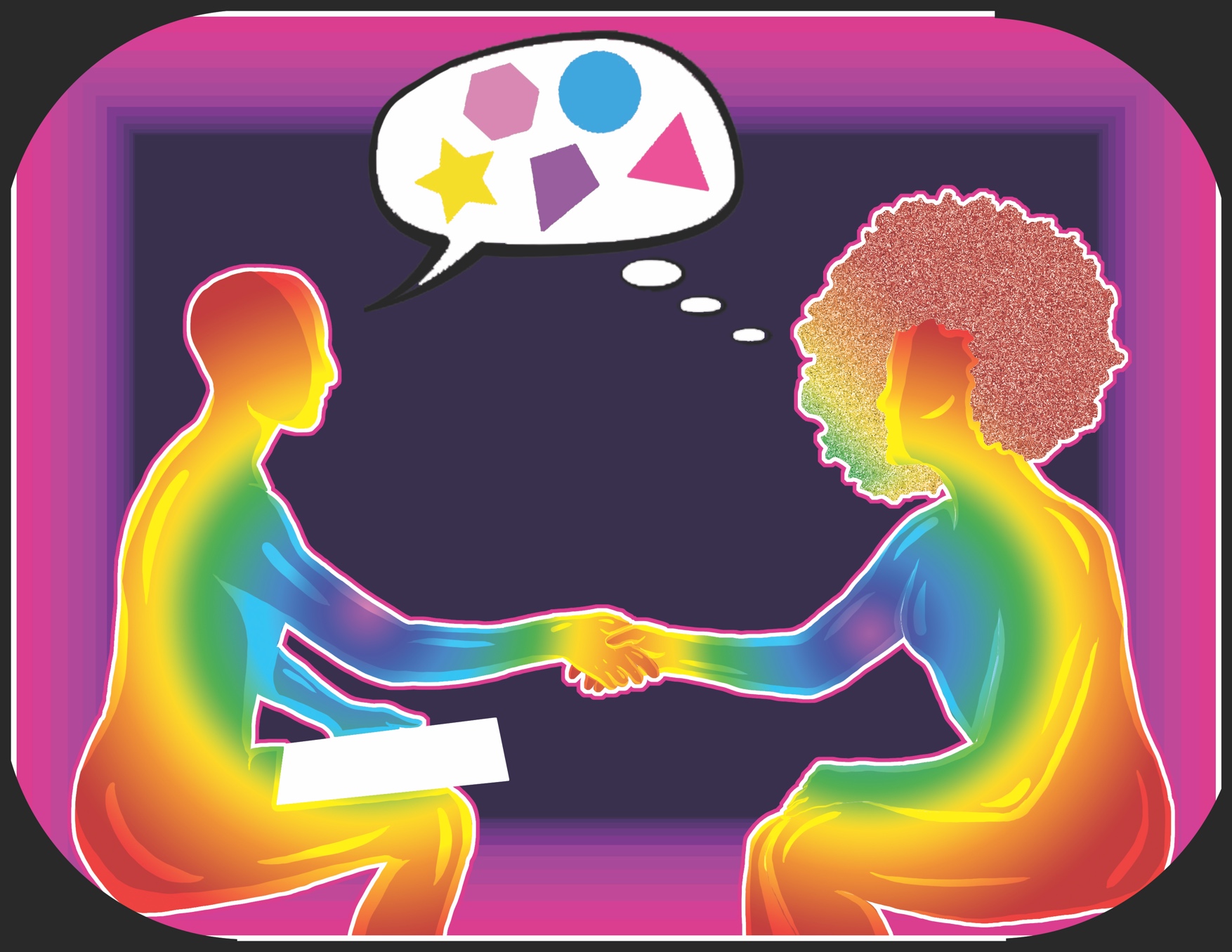
**
